# Transcranial Direct Current Stimulation for Improving Balance in Healthy Older Adults and Older Adults with Stroke: A Scoping Review

**DOI:** 10.3390/brainsci14101021

**Published:** 2024-10-15

**Authors:** Shih-Chiao Tseng, Sharon Dunnivan-Mitchell, Dana Cherry, Shuo-Hsiu Chang

**Affiliations:** 1 Department of Physical Therapy and Rehabilitation Sciences, University of Texas Medical Branch, Galveston, TX 77555-5302, USA; shdunniv@utmb.edu (S.D.-M.); dacherry@utmb.edu (D.C.); 2 Department of Physical Medicine and Rehabilitation, University of Texas Health Science Center at Houston, Houston, TX 77030-3870, USA; shuo-hsiu.chang@uth.tmc.edu

**Keywords:** tDCS, older adults, rehabilitation, falls, postural control

## Abstract

**Background/Objectives**: Age-related decline in balance and postural control is common in healthy elders and is escalated in aging adults with stroke. Transcranial direct current stimulation (tDCS) has emerged as one of the promising brain stimulations adjoining therapeutic exercise to enhance the recovery of balance and motor functions in persons with and without neurological disorders. This review aims to summarize and compare the available evidence of the tDCS on improving balance in the older adults without neurological disorders and the older adults with stroke. **Methods**: The Ovid (Medline) database was searched from its inception through to 06/15/2024 for randomized controlled trials investigating tDCS for improving balance in older adults with and without stroke. **Results**: Overall, 20 appropriate studies (including 271 stroke subjects and 259 healthy older adults) were found. The data indicate mixed results of tDCS for improving balance in older adults with and without stroke. **Conclusions**: Based on current research evidence, we have not found a specific tDCS protocol that is more effective than other tDCS protocols for improving balance and postural control in healthy older adults and older adults with stroke. Further research should explore the ideal tDCS approach, possibly in conjunction with standard interventions, to optimize postural control and balance in healthy older adults and older adults with stroke.

## 1. Introduction

Postural control, including static and dynamic balance, subserves all voluntary movements and prevents falls and injuries in the daily life [1,2]. According to U.S. Census, older adults aged 65 years and over are one of the fastest-growing populations in the country. Approximately 62 million adults aged 65 years and older live in the U.S., making up 18% of the population [3,4]. Furthermore, age is the significant risk factor for stroke [5]. Nearly 75% of all strokes occur in persons aged ≥65 years, and the prevalence of stroke doubles in adults over 75 years [6]. Balance or postural control deficits are highly prevalent in older adults and may be escalated in the aging adults with stroke, leading to increased mortality rate and disability, and decreased quality of life [7]. In 2018, more than 25% of older adults aged 65 years and older living in the U.S. reported at least one fall over past one year, and the risk of falling significantly increases in the elderly aged 85 years and older [8,9]. A recent statistic shows that the death rates due to unintentional falls are largely increased in older Americans during 1999–2020 [10]. Specifically, the older adults aged 85 years and older have the highest death rates (was 31.5% in 1999 and increased to 67.9% in 2020); the lowest death rates occurred among older adults aged 65–74 years (9% in 1999 and 18.2% in 2020, respectively) [10].

Transcranial direct current stimulation (tDCS) is a noninvasive, low-intensity brain stimulation technique that has been used to enhance balance and motor function in healthy adults and persons with neurological disorders [11,12,13,14,15,16,17,18,19,20]. The premise is that tDCS can modulate neural excitability and therefore enhance motor skill performance and learning [21,22,23,24,25]. The tDCS-induced modulation is polarity-dependent: the application of anodal tDCS (a-tDCS) increases neuronal excitation, whereas the application of cathodal tDCS (c-tCDS) decreases neuronal excitation over the region of interest [26,27,28,29,30]. In addition to polarity-dependent neural modulations, a recent system review suggests that the study population was a key factor in determining the therapeutic outcomes of tDCS on balance and postural control [31]. In addition, the effect of tDCS may show outcome-dependent effects. For example, tDCS application may enhance walking recovery but may not transfer to the improvements of balance or postural control [32,33]. Thus, this study aims to investigate and compare the available evidence of the tDCS on improving balance in the older adults without neurological disorders and the older adults with stroke. The data from healthy older adults without neurological disorders shed light on the impacts of tDCS on the normal, aging postural control system, whereas data from stroke populations provide additional insight on the aging postural system following stroke.

## 2. Materials and Methods

### 2.1. Search Strategy

**Figure 1:**
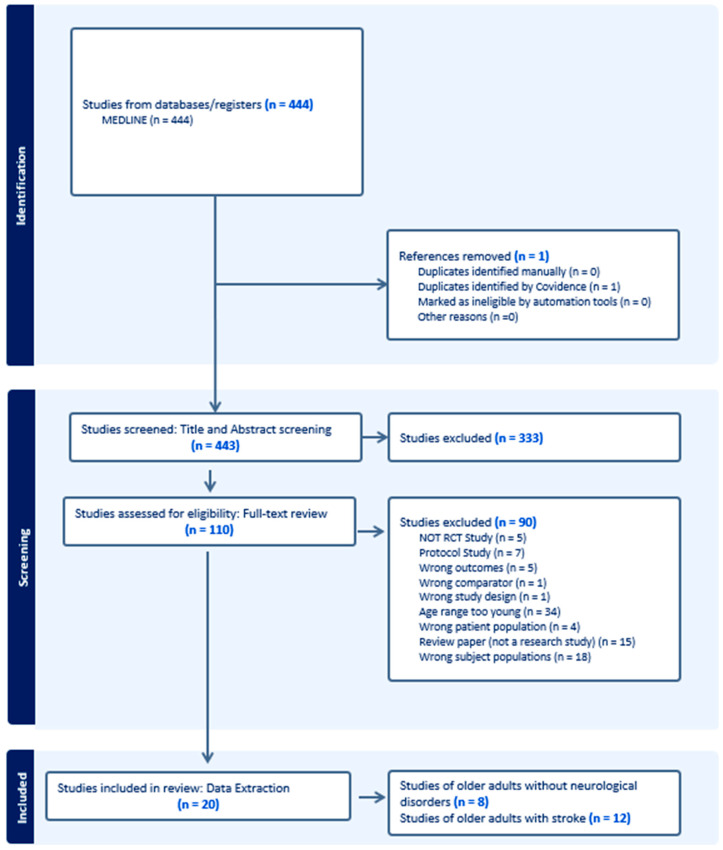
PRISMA flow diagram.

We conducted a systematic review using Preferred Reporting Items for Systematic Reviews and Meta-Analysis (PRISMA). The online database, OVID/Medline, was used for literature search. The search focused on studies that used tDCS to improve balance in older adults and older adults with stroke from inception to 15 June 2024. The following search terms were used: (1) “tDCS” or “a-tDCS” or “s-tDCS” and (2) “balance” or “postural control”, or “fall”, “frailty”, and (3) “older adults”, or “stroke”. Three independent reviewers (ST, SC, and SD) worked on two phases of the screening process, with the first phase being “title and abstract” screening, followed by the second phase, “full-text” screening (Figure 1). Any disagreements were resolved through discussion to reach consensus.

### 2.2. Inclusion and Exclusion Criteria

We used the population–intervention–comparison and outcome (PICO) approach to refine the search and define the scope of this review. The study populations consist of older adults without neurological disorders and older adults with stroke. The interventions involved tDCS alone or tDCS paired with exercise or skill training. The outcomes included static or dynamic balance and postural control or stability measures. Additional inclusion criteria were as follows: (1) randomized controlled trials, (2) crossover studies with the random assignments of tDCS protocols, (3) English publications, and (4) full-text articles available online in PDF or web (HTML) format. The exclusion criteria were as follows: (1) not human studies; (2) animal studies; (2) review articles; (3) case studies; (4) protocol studies; (5) books, theses, conference papers, commentaries, and letters; (6) studies that are not randomized controlled studies; (7) studies that do not have sham stimulation or control groups; and (8) study participants who are not healthy older adults or not older adults with stroke.

### 2.3. Data Extraction

Following the full-text screening, two researchers cross-checked all studies included in the review and extracted relevant data. Excel templates were used to prepare the standardized tables for data extraction, consisting of (1) the study author(s) and publication’s year, (2) study designs, (3) sample size and groups, (4) protocol setup and the parameters of tDCS (locations, types, intensities and durations), (5) total number of tDCS sessions, (6) additional exercise intervention adjoining tDCS, (7) timing of tDCS application relative to the exercise intervention, (8) outcome measures, and (9) primary findings related to the effects of tDCS interventions on balance outcomes. The authors resolved any inconsistencies in data extraction before data analysis.

## 3. Results

An online search from the database in MEDLINE identified 444 studies. Following the removal of duplicates, there was a total of 443 studies left. Title and abstract screening excluded 333 studies against the eligibility criteria, resulting in a total of 110 studies remaining for the next step of full-text screening. During full-text screening, another 90 studies were excluded, as outlined in Figure 1.

Twenty studies (12 focusing on older adults with stroke and 8 on older adults without neurological disorders) met the eligibility criteria and were included in this review [11,12,14,15,18,20,32,33,34,35,36,37,38,39,40,41,42,43,44,45]. The included studies were categorized into two groups: older adults with stroke and older adults without neurological disorders. The salient characteristics of these studies are outlined in Table 1 and Table 2. The included studies constituted a total of 530 older adults, including 271 older adults with stroke and 259 older adults without neurological disorders.

**Figure 2:**
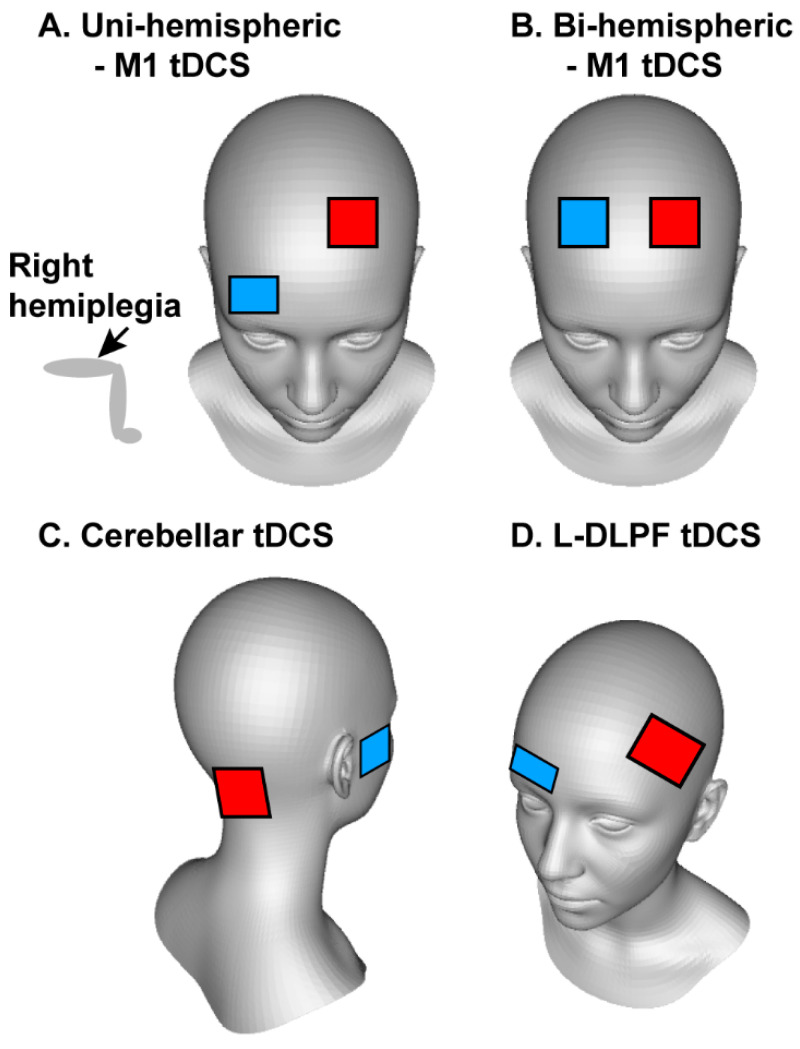
Electrode placements for different transcranial direct current stimulation (tDCS) applications. (**A**) Uni-hemispheric primary motor cortex (M1) tDCS. (**B**) Bi-hemispheric M1 tDCS. (**C**) Cerebellar tDCS. (**D**) Left dorsal lateral prefrontal (L-DLPF) tDCS. Red square is the electrode for anodal (i.e., active) stimulation. Blue square is the electrode for cathodal or reference electrode. During uni-hemispheric M1 tDCS stimulation, the anodal electrode was placed on the leg area of the ipsilesional M1 while the reference electrode was placed on contralateral supraorbital area (**A**). During bi-hemispheric M1 tDCS stimulation, the anodal electrode was placed on the leg area of the ipsilesional M1 while the cathodal electrode was placed on contralesional M1 (**B**). During cerebellar stimulation, the anodal electrode was placed on the median line 1–2 cm below the inion of the occipital bone and the reference electrode was placed on the right buccinator muscle (**C**). During L-DLPF stimulation, the anodal electrode was placed on the L-DLPF area and the reference electrode was placed on contralateral supraorbital area (**D**).

Interestingly, our review revealed a notable distinction in tDCS applications for older individuals with and without stroke. For older adults with stroke, a-tDCS was predominantly focused on the ipsilesional primary motor cortex (M1), while in healthy older adults, the stimulation was primarily directed towards the left dorsal lateral prefrontal cortex (L-DLPFC) or cerebellum (Figure 2). A brief comparison of the stroke and healthy adult studies is presented below.

### 3.1. Stroke tDCS Studies (12 Studies)

Ten out of twelve stroke studies applied a-tDCS over the ipsilesional M1 (Table 1). The remaining two studies used tDCS on the ipsilesional supplementary motor area (SMA, n = 1) or the ipsilesional or contra-lesional cerebellum (n = 1). Half of the 12 stroke studies utilized a crossover within-subject design which had a single a-tDCS session [20,32,34,35,36,46]. The other half were interventional studies, involving concurrent application of a-tDCS and therapeutic exercise or skill training over multiple sessions [11,15,18,33,37,38]. There was a trend toward greater improvements in balance and postural control when a-tDCS was combined with multiple sessions of task-specific training (ex. gait or balance training) for persons with stroke [15,18,38]. Although half of the interventional studies found a-tDCS to be more effective than sham tDCS in improving balance or postural control, crossover studies indicated limited benefits from a single a-tDCS session.

In addition, two studies examined the effects of bi-hemispheric tDCS applications over bilateral M1 areas on postural and balance recovery in persons with stroke [32,38]. This stimulation involved anodal stimulation over the affected M1 and cathodal stimulation over the unaffected M1. Youssef et al. (2023) [38] compared uni-hemispheric versus bi-hemispheric tDCS protocols and found no significant differences between both approaches in enhancing balance in persons with stroke.

### 3.2. Healthy Older Adult tDCS Studies (8 Studies)

Unlike stroke studies, tDCS research on healthy older adults primarily focused on the left dorsolateral prefrontal cortex (L-DLPFC, four studies) and bilateral cerebellum (four studies, Table 2). Three studies were crossover studies which had a single a-tDCS session [14,43,45]. The other five studies were interventional studies which combined multiple training sessions and tDCS [12,40,41,42,44]. Aligned with stroke research, a single-session tDCS had minimal impact on postural or balance improvements; repeated tDCS sessions, especially when paired with task-specific training, may be more beneficial for improving balance in older adults.

In addition, for healthy older adults, bilateral cerebellar a-tDCS [40,42,44] was better than L-DLPFC stimulation in improving postural control and balance [12,41,43,45]. There was no significant difference between cerebellar and left M1 stimulation [14,44].

## 4. Discussion

This review aimed to assess the effectiveness of transcranial direct current stimulation (tDCS) in enhancing postural control and balance among older adults. Our findings revealed varying tDCS protocols for healthy older adults and those with stroke. While L-DLPFC and cerebellar a-tDCS were common targets for healthy individuals, ipsilesional M1 was primarily stimulated in stroke patients. However, current research does not support the efficacy of any specific tDCS protocol for improving balance and postural control in either group.

### 4.1. Effects of tDCS on Postural Control and Balance in Older Adults with Stroke

Growing research indicate that applying a-tDCS to the affected side of the brain in persons with stroke patients can enhance motor learning and functional recovery, including balance and walking [31,47,48]. Two common electrode placements, or montages, used to increase neuronal activity in the damaged M1 were uni-hemispheric M1 tDCS and bi-hemispheric M1 tDCS. For uni-hemispheric M1 tDCS, the anodal (i.e., active) electrode was placed over the lesioned M1 to enhance cortical excitation, while the reference electrode was positioned over the contralesioned supraorbital area. For bi-hemispheric M1 tDCS application, the anodal/active electrode was placed over the injured M1 to increase neuronal excitation while the cathodal electrode was placed over the intact M1 contralateral to the lesioned M1 to reduce transcallosal inhibition. Although research on stroke survivors has yielded inconsistent findings regarding the effectiveness of unilateral versus bilateral M1 tDCS, a recent meta-analysis suggests bilateral tDCS may be more effective than unilateral stimulation [48]. However, this conclusion is primarily supported by a single study with larger effect size for bilateral M1 tDCS [49].

This systematic review found that the uni-hemispheric M1 a-tDCS is the most common intervention for improving balance and postural control in older adults with stroke. Yet, only one-third of studies (33%) demonstrated a significant advantage of the uni-hemispheric M1 a-tDCS over sham tDCS [18,36,38]. Compared to previous research, uni-hemispheric M1 a-tDCS appears to be less effective in improving balance and postural control in this population. Our review of uni-hemispheric versus bi-hemispheric M1 tDCS for improving postural control and balance in older adults with stroke yielded mixed results. While Youssef et al. (2023) found both approaches to be effective for improving balance compared to sham tDCS [38], Saeys et al. (2015) did not observed significant differences in balance scores between bi-hemispheric M1-tDCS and sham groups [32]. Given these conflicting findings, more research is needed to determine the optimal approach for this population.

Our review found that only two studies have applied a-tDCS on non- M1 regions in older adults with stroke: one was over the cerebellum [39], and the other one was over ipsilateral supplementary motor area (SMA) [35]. These studies suggest that a-tDCS over the cerebellum and SMA could improve static and dynamic balance for older adults with chronic stroke [35,39]. The evidence suggests that cerebellar and SMA a-tDCS could enhance motor planning and learning, leading to greater improvements in postural control and balance for persons with stroke [35,39,50]. A recent systematic review found both cerebellar a-tDCS and M1 a-tDCS can enhance motor recovery in persons with stroke [50]. More research is needed to determine if cerebellar tDCS can help older stroke patients improve their balance and posture.

### 4.2. Effects of tDCS on Postural Control and Balance in Healthy Older Adults

Our review identified L-DLPFC and cerebellar tDCS as the primary targets for a-tDCS application for improving postural control and balance in healthy older adults. Among four studies targeting L-DLPFC [41,43,45,51], only one study demonstrated a reduction in dual-task costs for postural sway compared to the sham applications [45]. Three out of four studies investigating cerebellar a-tDCS in older adults reported significant improvements in postural control or balance, particularly among those at high risk of falls [40,42,44]. The findings suggest that cerebellar a-tDCS could be a promising intervention for enhancing postural control and balance in healthy older adults.

Additionally, this review revealed that a single tDCS session had minimal benefits on postural or balance improvements. Multiple tDCS sessions, particularly when combined with task-specific training, could be more effective in improving postural control and balance in both healthy older adults and older adults with stroke.

### 4.3. Limitations and Recommendations

To our knowledge, this is the first review focusing on the effects of tDCS on postural control and balance in both healthy older adults and older adults with stroke. Our review found insufficient evidence to conclusively determine the effectiveness of tDCS in improving postural control and balance in older adults. The heterogeneity in study methods and designs is a limitation that could potentially bias the results. The therapeutic interventions used in conjunction with tDCS varied widely, confounding the balance outcomes. Another limitation is that many tDCS studies had small sample sizes, limiting their statistical powers and potentially leading to biased conclusions. Future clinical trial studies should include larger samples of older adults. Additionally, follow-up assessments should be conducted to evaluate the long-term effects of tDCS on postural and balance in older adults.

## 5. Conclusions

This scoping review offers a preliminary examination of tDCS’s potential to enhance balance and postural control in older adults. Tailored tDCS protocols are likely required for older adults with and without neurological conditions. Current evidence indicates that different tDCS protocols are needed for older adults with and without neurological disorders. To determine the effectiveness of various tDCS protocols for balance and postural control in older adults, large-scale clinical trials are essential. These studies should compare outcomes between healthy individuals and those with neurological conditions like stroke or Parkinson’s disease. Enhancing balance in older adults, regardless of neurological status, is vital for improving their quality of life, functional independence, and reducing risks of falling and mortality rate. Further research should explore the ideal tDCS approach, possibly in conjunction with standard interventions, to optimize postural control and balance in healthy older adults and older adults with stroke. This will contribute to more effective rehabilitation strategies and guide clinical practice.

## Figures and Tables

**Table 1:** Characteristics of included studies for older adults with stroke.

Authors	Study Design	Blinding	Sample	tDCS Electrode		tDCS Parameters	Number of Sessions	Additional Activity	Time of tDCS Relative to Activity	Balance Outcome Measure	Time of Assessment	Main Results
				Active/ Anode	Reference/ Cathode							
Chang et al., 2015 [11]	Parallel group	double-blind	Subacute stroke (n = 24): anodal (n = 12) sham (n = 12)	ipsilesional M1-TA	contra-lateral supraorbital area	2 mA, 10 min	10 sessions	Conventional PT	during	BBS	pre- and post- intervention	No significant differences in BBS between groups after a 2-week intervention.
Ehsani et al., 2022 [15]	Parallel group	Double-blind	chronic stroke (n = 32): anodal (n = 12) sham (n = 10) control (n = 10)	ipsilesional M1	contra-lateral supraorbital area	1 mA, 20 min	10 sessions	Conventional PT	during	BBS	Pre-, post- and one month after intervention	BBS was significantly improved in the anodal group immediately and 1 month after intervention. No significant improvements in BBS were found in other two groups.
Fruhauf et al., 2017 [34]	Crossover	double-blind	chronic stroke (n= 30): anodal + FES (n = 30) sham + FES (n = 30) anodal + sham FES (n = 30) sham + sham FES (n = 30)	ipsilesional M1	contra-lateral supraorbital area	2 mA, 20 min	One for each STIM condition	Active or Sham FES on TA for 20 min	during	Postural sway	before and immediate after each STIM condition	The combination of anodal tDCS and active FES had no significant improvements of static balance in persons with chronic stroke.
Kim et al., 2024 [18]	Parallel group	Double-blind	chronic stroke (n = 24): anodal (n = 12) sham (n = 12)	ipsilesional M1	ipsilateral to anodal SITM	2 mA, 30 min	10 sessions	Robotic assisted gait training (RAGT)	during	TUG, BBS	Pre- and post-intervention; pre- and one-month follow-up	Both groups showed significant improvements in BBS and TUG after the intervention. not described on extraction. At one-month follow-up, a-tDCS group showed significant improvements in both BBS and TUG whereas s-tDCS showed significant changes only in TUG.
Liang et al., 2020 [20]	Crossover	double-blind	chronic stroke (n= 10): anodal (n = 10) sham (n = 10)	ipsilesional M1	contra-lateral supraorbital area	2 mA, 20 min	One for each STIM	Postural training (limits of stability)	during	BBS	Pre- and post- intervention	No significant changes in BBS were found between groups and at post-intervention compared to pre-intervention. Postural sway was reduced at similar levels between two groups after intervention
Manji et al., 2018 [35]	Crossover	double-blind	chronic stroke (n = 30): anodal (n = 30) sham (n = 30)	ipsilesional SMA	inion	1 mA, 20 min	One for each STIM	BWSTT	during	TUG	Pre- and post- intervention	Significant improvements of TUG scores were found in a-tDCS group compared to the s-tDCS group.
Saeys et al., 2015 [32]	Crossover	double-blind	subacute stroke (n = 31): anodal (n = 31) sham (n = 31)	ipsilesional M1	contra-lesional M1	1.5 mA, 20 min	16 sessions	PT and OT	before	Tinetti Balance score	Pre- and post-intervention, 4- and 8- week follow-up	Tinetti balance scores were significantly improved in both groups after intervention. There were no group differences in Tinetti balance scores.
Seo et al., 2017 [33]	Parallel group	double-blind	chronic stroke (n = 21): anodal (n = 11) sham (n = 10)	ipsilesional M1	contra-lateral supraorbital area	2 mA, 20 min	10 sessions	robotic-assisted gait training (RAGT)	before RAGT	BBS	Pre- and post-intervention, 4-week follow-up	No group differences in the improvements of BBS.
Sohn et al., 2013 [36]	Crossover	single-blind	chronic stroke (n = 11): anodal (n = 11) sham (n = 11)	ipsilesional M1	contra-lateral supraorbital area	2 mA, 10 min	One for each STIM	No	N/A	Static balance	Pre- and post- intervention	The a-tDCS group showed significant improvements of postural stability after intervention compared to s-tDCS group.
Toktas et al., 2024 [37]	Parallel group	double-blinded	chronic stroke (n = 28): anodal (n = 14) sham (n = 14)	ipsilesional M1	contra-lateral supraorbital area	2 mA, 20 min	20 sessions	task- oriented physiotherapy	concurrent	BBS, TUG	Pre- and post- intervention	There were significant improvements of TUG and BBS after intervention in both groups. There were no group differences in balance improvements.
Youssef et al., 2023 [38]	Parallel group	double-blind	subacute stroke (n = 35): anodal-bi-hemispheric (n = 11) anodal-uni-hemispheric (n = 13) sham (n = 11)	ipsilesional M1	contra-lesional M1 or contra-lateral supraorbital area	2 mA, 20 min	12 sessions	physiotherapy	during	BBS	Pre- and post- intervention	Both uni-hemispheric and bi-hemispheric anodal stimulation significantly improved balance more than sham stimulation.
Zandvliet et al., 2018 [39]	Crossover	single-blind	chronic stroke (n = 15): contra-lesional cerebellar, ipsi-lesional cerebellar, or sham healthy adults (n = 10): contra-lesional cerebellar or ipsilesional cerebellar	ispilesional or contra-lesional cerebellum	ipsilateral buccinators muscles	1.5 mA, 20 min	one for each STIM	postural tracking task	during	COP	Pre- and post- intervention	There was significant decrease in CoP only in tandem stance for the stroke group after contra-lesional stimulation. There were no significant differences in CoP between sham and cerebellar stimulations for healthy adults.

Note: Abbreviations: tDCS: transcranial direction current stimulation; a-tDCS: anodal transcranial direct current stimulation; M1: primary motor cortex; SMA: supplementary motor area; STIM: stimulation; PT: physical therapy; OT: occupational therapy; BWSTT: body-weight support treadmill training; BBS: Berg Balance Score; TUG: Time up and Go test; COP: center of pressure; N/A: not applicable.

**Table 2:** Characteristics of included studies for older adults without neurological disorders.

Authors	Study Design	Blinding	Sample	tDCS Electrode		tDCS Dose	Number of Sessions	Additional Activity	Time of tDCS Relative to Activity	Outcome Measure	Baseline Balance Score	Time of Assessment	Main Results
				Active/ Anode	Reference/ Cathode								
Correa et al., 2023 [12]	Parallel groups	triple-blind	older adults (n = 28) (1) anodal (n = 14) (2) sham (n = 14)	Left DLPC	contra-lateral supraorbital area	2 mA, 20 min	24	Multi-component training (MT)	during MT	Mini BESTest	Anodal: 26 ± 5 Sham: 24 ± 5	pre- and post-intervention, 30 day follow-up	There were no significant differences in MiniBEST scores between groups after intervention and at 30-day follow-up
Craig et al., 2017 [14]	Crossover	double-blind	older adults (n = 18): (1) M1 a-tDCS (n = 18) (2) cerebellar a-tDCS (n = 18) (3) sham M1 (n = 18)	M1 or median line 2 cm below the inion	inion or right buccinator muscles	2 mA, 20 min	One for each STIM	Postural control task (PCT)	during PCT	Postural sway	none	pre-. Post-, 30 min post	There were no significant differences in postural sway between stimulation conditions.
Ehsani et al., 2017 [40]	Parallel groups	double-blind	older adults (n = 29): (1) cerebellar a-tDCS (n = 14) (2) sham (n = 15)	1 cm below inion of occipital bone	right arm	1.5 mA, 20 min	1	none	N/A	BBS	Anodal: 42.45 ± 1.43 Sham: 42.91 ± 1.87	pre- and post-intervention, 48 h follow-up	Improved postural stability and BBS were found after cerebellar a-tDCS intervention.
Lo et al., 2023 [41]	Parallel groups	double-blind	older adults (n = 6) (1) DLPC a-tDCS (n = 2) (2) sham (n = 4)	Left DLPC	not specified	<4 mA, 20 min	up to 10	Conventional PT	Before PT	BBS, TUG	BBS scores: Anodal: 44 ± 1.41 Sham:41.25 ± 5.97	pre- and post- intervention	There were no significant differences in balance improvements between groups.
Parsaee et al., 2023 [42]	Parallel groups	single-blind	older adults (n = 24) (1) cerebellar a-tDCS (n = 12) (2) sham (n = 12)	Inion	left eye socket	2 mA, 15 min	3	none	N/A	TUG, static balance	TUG scores: Anodal: 11.29 ± 1.07 sham: 11.28 ± 0.98	pre- and post- intervention	The cerebellar a-tDCS group showed significant improvements in static balance and TUG after intervention compared to sham group
Schneider et al., 2021 [43]	Crossover	double-blind	older adults (n = 25): (1) DLPC a-tDCS + walking (n = 25) (2) DLPC a-tDCS + seated (n = 25) (3) sham + walking (n = 25)	Left DLPFC + M1	Left PF and left parietal lobe	3 mA, 20 min	1 for each STIM	N/A	N/A	Postural sway	not reported	pre- and post- intervention	There were no significant improvements of postural sway after a-tDCS stimulations.
Yosephi et al., 2018 [44]	Parallel groups	double-blind	Older adults (n = 62): (1) cerebellar a-tDCS+ postural training (n = 12) (2) left M1 a-tDCS + postural training (n = 13) (3) sham tDCS + postural training (n = 12) (4) postural training (n = 12) (5) cerebellar a-tDCS (n = 13)	(1) cerebellum (1 cm below inion of occipital bone) (2) left M1 (3) cerebellum or left M1 (randomized)	(1) right buccinator muscles (2) right contralateral supraorbital area (3) right buccinator muscles or right contralateral supraorbital area	2 mA, 20 min	6	Postural training	during postural training	BBS, static balance	BBS: (1) 39.55 ± 1.43; (2) 39.58 ± 1.38; (3) 39.50 ± 0.87; (4) 38.18 ± 1.01; (5) 38.84 ± 0.91	pre- and post- intervention	Postural training with M1 or cerebellar a-tDCS, especially cerebellar atDCS, can significantly improve postural control or balance in older adults with high fall risks after two-week intervention. Cerebellar a-tDCS alone is not a sufficient intervention.
Zhou et al., 2021 [45]	Crossover	double-blind	older adults (n = 57): (1) DLPC + SM1 a-tDCS (n = 12) (2) DLPC a-tDCS (n = 14) (3) SM1 a-tDCS (n = 16) (4) sham tDCS (n = 15)	Left DLPFC, SM1	Left PF, Temporal, and Occipital lobes	<4 mA, 20 min	1 session for each STIM	none	N/A	TUG, postural sway	TUG: 12 ± 3	pre- and post-stimulation	The DLPFC + SM1 and DLPFC a-tDCS groups had lower dual-task costs to postural sway compared to the SM1 or sham groups.

Note: Abbreviations: tDCS: transcranial direction current stimulation; a-tDCS: anodal transcranial direct current stimulation; DLPFC: dorsal lateral prefrontal cortex; M1: primary motor cortex; SM1: primary sensorimotor area; STIM: stimulation; PT: physical therapy; OT: occupational therapy; BWSTT: body-weight support treadmill training; BBS: Berg Balance Score; TUG: Time up and Go test; N/A: not applicable.

## Data Availability

Data are included in this study.
